# Mate Choice in Western Mosquitofish (*Gambusia affinis*) in Response to Virtual Mates: A Method for the Investigation of Fish Mate Choice Using Maya 3D Simulation Technology

**DOI:** 10.3390/ani14233369

**Published:** 2024-11-22

**Authors:** Bowen Feng, Liming Chen, Liangmin Huang, Jun Li, Kai Liu

**Affiliations:** 1 Fisheries College, Jimei University, Xiamen 361021, China; fengbowen0323@163.com (B.F.); chen1015085915@163.com (L.C.); lmhuang@jmu.edu.cn (L.H.); lijun1982@jmu.edu.cn (J.L.); 2 Fujian Provincial Key Laboratory of Marine Fishery Resources and Eco-Environment, Fisheries College, Jimei University, Xiamen 361021, China; 3 State Key Laboratory of Mariculture Breeding, Fisheries College, Jimei University, Xiamen 361021, China

**Keywords:** mate choice, Maya 3D simulation animation, visual signals, *Gambusia affinis*

## Abstract

Visual signals are crucial for animals to obtain information about their environment, and they play a significant role in mate choice. However, individual variability and factors such as movement patterns can hinder research flexibility. A key challenge in this field is the accurate simulation of specific movements and behaviors. In this study, we investigated the western mosquitofish (*Gambusia affinis*) by creating 3D simulation animations using the Maya 2018 software. Meanwhile, we validated the effectiveness of this animation through dichotomous association preference tests. The results showed that our subjects could successfully identify 3D simulated mates, and both males and females demonstrated pronounced preferences for larger simulated animations. Moreover, our findings revealed that this species exhibited a notably stronger preference for 3D simulations compared to 2D animations. These findings suggest that 3D simulation technology holds significant potential for the investigation of fish mate choice, offering an efficient, precise, standardized, and easily manageable non-invasive method for future research in fish behavior.

## Simple Summary

This study explored how visual signals affect mate choice in animals, focusing on the western mosquitofish (*Gambusia affinis*). We created 3D simulation animations using the Maya 2018 software to accurately replicate key movements and behaviors essential for our research. Through preference tests, we validated these animations and found that the fish could effectively identify 3D simulated mates. Both male and female mosquitofish exhibited a strong preference for larger animations. Additionally, the fish displayed significantly greater attraction to 3D simulations compared to 2D ones. These findings underscore the potential of 3D simulation technology in studying fish behavior, offering an efficient, precise, and non-invasive method for future research on mate choice. This work not only enhances our understanding of animal behavior but also contributes to conservation strategies aimed at preserving fish populations and their ecosystems.

## 1. Introduction

Visual signals play a crucial role in many species within the animal kingdom, particularly in aspects such as mate selection, foraging, and predator avoidance [1,2,3,4]. Studies have shown that fish can detect visual signals from predators and adjust their behavior in territory defense, the evasion of threats, and courtship activities [5,6]. For example, juvenile coho salmon (*Oncorhynchus kisutch*) exhibited a significant reduction in aggressive behavior towards mirror images when exposed to visual signals from avian predators [7]. Mate choice is vital to sexual selection and biological evolution [8,9,10], influencing many species’ reproductive success and population dynamics. It involves a range of complex and variable signals, including size, coloration, distinctive ornamental features, and movement patterns, which are often dynamic and change over time and space [11,12,13,14]. In fish, mate choice is typically driven by visual, chemical, and auditory cues, with visual signals playing a critical role in many species. Currently, many methods in fish mate choice studies are based on visual signals [15]. Utilizing these visual characteristics, researchers have developed various approaches for investigation, including models, images, and video playback [16,17,18,19].

With the advancement of computer imaging technology, an increasing number of studies have employed computer-simulated animations in the research of fish behavior, successfully applying this technique to investigate mate choice in various social animals, such as three-spined sticklebacks (*Gasterosteus aculeatus*) [20], pipefish (*Syngnathus typhle*) [21], zebrafish and their allies (genus *Danio*) [22,23], and pygmy swordtails (*Xiphophorus nigrensis*) [24]. Two-dimensional (2D) simulation animation technology has been widely utilized to explore fish mate choice preferences. For instance, in studies on mate selection in the striped cichlid (*Pelvicachromis taeniatus*), it was found that the stimulatory effects of 2D simulation animations outperformed those of static images [25]. Two-dimensional simulation animations offer several advantages in studying fish mate choice, including preventing habituation due to the stimuli’s movement characteristics, maintaining subjects’ attention, consistently eliciting social responses, and ease of production. However, this technology still has limitations in terms of animation fidelity and realism. For example, fish’s nuanced and flexible behaviors during swimming and courtship, such as turning, fin waving, and reproductive fin display, are challenging to accurately represent using traditional 2D simulation animations. Therefore, adopting a method with higher simulation fidelity and more precise behavioral characteristics would greatly enhance the study of fish mate choice behaviors.

As three-dimensional (3D) simulation animation technology advances, the use of 3D simulation animations in the study of animal behavior has matured, offering an innovative visual presentation method to simulate, analyze, and showcase the natural behaviors of animals and their habitats [26]. This technology enables the creation of complex animal behaviors and interaction scenarios without disturbing the animals or their living environments [27]. Compared to 2D simulation animations, 3D simulation animations possess unique advantages, enhancing their applicability in specific contexts. Firstly, 3D simulation animations provide a realistic sense of depth and space, offering experimental subjects a more immersive experience. Secondly, 3D simulation animations can produce highly realistic textures, lighting effects, and surface details through advanced rendering techniques, improving the visual quality. Recently, 3D simulation animation technology has been employed in animal behavior research by establishing 3D simulation animated models, yielding positive results. For example, in studies examining how the jumping spider (*Portia fimbriata*) responds to visual cues in its prey’s eyes, researchers used computer-rendered virtual 3D lures to manipulate these lures and investigate the spider’s hunting behavior and decision-making processes based on visual information from the prey’s eyes [28]. Additionally, to study the key role of visual signals during lizard movement, the Maya 3D simulation animation software was used to simulate the movement of individuals in a three-dimensional space, representing an innovative approach to understanding dynamic environments that merges evolutionary biology with digital art, demonstrating the method’s practicality through the examination of lizard movements in a plant motion noise environment [29,30]. However, the full potential of 3D simulation animations in fish behavior research remains underutilized, with only a limited number of studies reported. For instance, researchers created 3D simulation animations of the swimming behavior of *Oryzias latipes*, establishing a solid technical foundation for the application of 3D simulation animations in fish behavior studies [31]. Furthermore, in research on mate choice in *Poecilia latipinna*, animations constructed using the Blender 3D software demonstrated that the simulation intensity could match that of live animals [32,33]. In a study examining the mate choice of male *Etheostoma zonale* for conspecific versus heterospecific females of *E. barrenense*, the researchers found no significant difference in the mate preference intensity between live heterospecific individuals and animated simulations [34]. Overall, 3D simulation animations offer high manipulability and standardized approaches for fish behavior research, with the potential to reduce and replace live animal stimuli while optimizing the experimental protocols. Therefore, effectively utilizing 3D simulation animations to study fish behavior is a worthwhile area for exploration.

This study focuses on the western mosquitofish (*Gambusia affinis*), investigating its mate choice behavior when exposed to 3D simulation animations. Previous studies on this species have highlighted several factors influencing its mate choice, including its body size, coloration, courtship behavior, and male ornamentation [35,36]. Prior research has demonstrated high consistency between the mate choice behaviors of live *G. affinis* and those in computer-animated experiments, with both males and females showing apparent mating behaviors towards animated partners, thereby validating the effectiveness of 2D simulation animations in mate choice studies for this species [37]. Hence, we aim to create an animated model of *G. affinis* using the Maya 3D technology to comprehensively examine both sexes’ morphological and behavioral characteristics and explore the potential of 3D simulation animations in mate choice research. We hypothesize that (1) *G. affinis* can effectively recognize 3D simulation animated mates, and (2) compared to 2D simulation animations, 3D simulation animations offer a greater advantage in studying mate choice in *G. affinis*.

## 2. Materials and Methods

### 2.1. Origin and Maintenance of Test Fish

All test fish used in this study were wild-caught individuals collected from the field area of Longyan City, Fujian Province, China (N 25°05.33′, E 117°00.32′; Figure S1). We placed the collected *G. affinis* in 200 L transparent glass tanks equipped with an automatic oxygen supply and a circulating filtration system. The bottom of each tank was layered with gravel, aquatic plants, and stones to simulate a natural environment. A 12-h light/12-h dark cycle was maintained, and each tank housed 40 experimental fish with a 1:1 male-to-female ratio for an acclimation period of 21 days. The fish were fed twice daily (at 09:00 and 17:00) with commercially available flake food and frozen bloodworms. The water temperature in both the holding and experimental tanks was maintained at 25 ± 1 °C. We maintained the water quality by exchanging half of the water in each holding tank every two weeks, using filtered tap water throughout the experiment [37].

### 2.2. Three-Dimensional Simulation Animation Modeling

#### 2.2.1. Images Collection

We collected high-resolution images of *G. affinis*, following Chen et al. [37]. To create 3D simulation animations that accurately reflected the natural variations in the body size and swimming speed of the subjects, we anesthetized the fish using a 10% clove oil solution after photographing (Sony a7R III Mirrorless, Sony, Tokyo, Japan) them, and we measured the standard length (SL) of 30 wild *G. affinis* individuals (15 females and 15 males). Additionally, the swimming speed of another 30 individuals (15 females and 15 males) was assessed using an activity measurement method [37]. Finally, image data of 15 females and 15 males *G. affinis* were collected (mean ± SD, females SL: 28.49 ± 3.3 mm, *n* = 15; male SL: 21.83 ± 2.66 mm, *n* = 15; Figure S2), along with video data from 10 individuals (5 females and 5 males; female swimming speed: 2.96 ± 0.64 cm/s, *n* = 5; male swimming speed: 2.81 ± 0.88 cm/s, *n* = 5). These data were used to create the 3D simulation animations.

#### 2.2.2. Modeling

**Figure 1:**
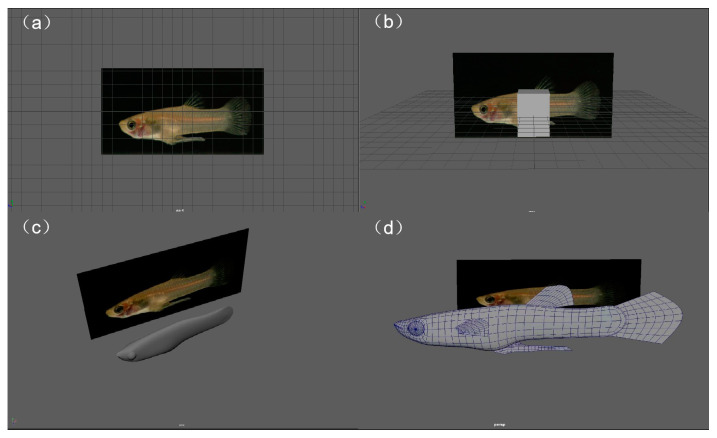
The modeling procedure. (**a**) Import reference image. (**b**) Add cube. (**c**) Fit fish body contour. (**d**) *G. affinis* model.

The 3D simulation animation models were created using Autodesk Maya 2018 (Autodesk, San Francisco, CA, USA) for 3D graphics, Adobe Premiere Pro 2020 (Adobe, San Francisco, CA, USA) for video processing, Adobe Substance 3D Painter 7.4.1.1418 (Adobe, San Francisco, CA, USA) for 3D model texturing, and Adobe Photoshop 2020 for image editing. First, an initial model was established in Maya, using a high-resolution image of *G. affinis* as a reference image (Figure 1a). A cube (polycube) was added as the basic structure of the model (Figure 1b). The model was then switched to Edit Mode, where tools such as Rotate, Extrude, and Cut were used to create and adjust new vertices. These vertices were moved to fit the fish’s external contours, as shown in the reference image (Figure 1c). Next, mirroring was performed along the X, Y, or Z axis of the scene to create a symmetrical half of the head, and a mirror command was used to generate the mirrored geometry, which was then merged with the other side to complete the body model. Finally, the Smooth command was used to smooth the body, completing the model’s construction (Figure 1d) [38].

#### 2.2.3. UV Unfolding and Mapping

**Figure 2:**
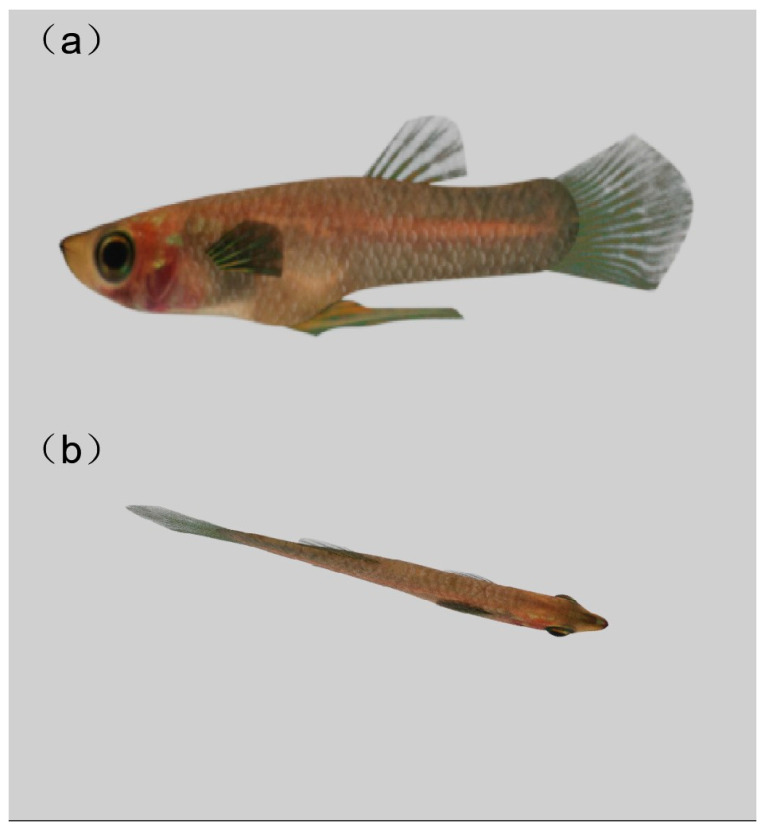
The model mapping. (**a**) Model (side view). (**b**) Model (top view).

To create the UV mapping for the fish model, we first selected the geometric components of the fish body using the model’s component mode. An appropriate axis, typically the *Z*-axis, was then chosen to establish an initial planar mapping, ensuring that the entire fish body was encompassed within the UV (U-VEEZ) space. Next, we utilized the Cut UV Edges tool in the UV editor to sever the UV shell along the edges of the fish body, thereby minimizing UV stretching and distortion. Subsequently, we selected the Automatic option from the UV menu to enable Maya to automatically unfold the UV shell. Afterward, the fish body UV shell was selected, ensuring that it remained within the 0 to 1 UV space without overlapping. Manual adjustments to the position and scale of the UV shell were made to guarantee even texture distribution. The UV unfolding process for the fish’s eyes and fins followed the same methodology. Then, the image editing software (e.g., Substance 3D Painter 7.4.1.1418) was employed to paint the textures for the fish body, eyes, and fins based on the UV layout. The painted texture files were then imported into Maya and applied to the model, verifying the texture fitting. An Arnold material was subsequently created and applied to the model within the material properties. These steps were repeated to generate materials and apply textures for the fish’s eyes and fins. Finally, Maya’s Arnold rendering engine was utilized for a preview render (Figure 2) [38].

#### 2.2.4. Skeletal Binding

**Figure 3:**
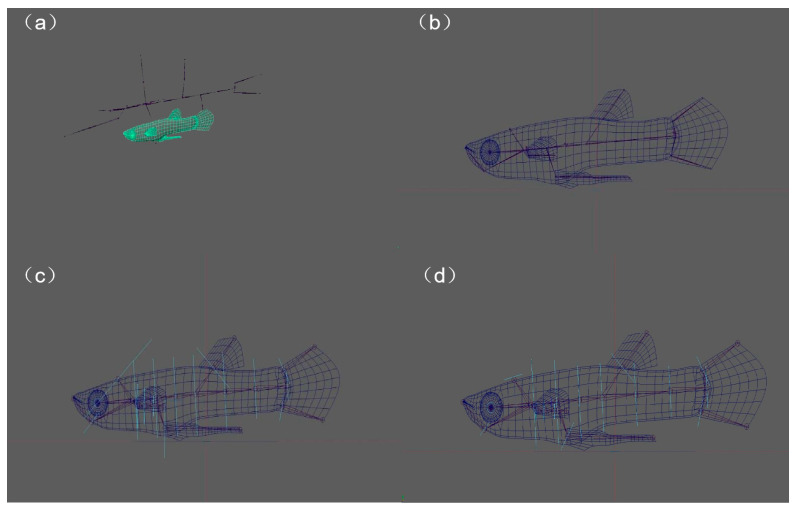
Skeletal binding. (**a**) Create bones. (**b**) Position bones. (**c**) Create controllers. (**d**) Adjust controllers.

We initiated the process by accessing the tool panel and selecting the “Fish Structure” option to create a basic skeletal chain using the Advanced Skeleton menu in Maya (Figure 3a). The generated skeletal chain was subsequently adjusted to align with the fish’s anatomical structure. The Move tool was utilized to manually position each bone, ensuring alignment with the respective components of the fish model, including the head, spine, and fins. The Orient Joint tool was then employed to guarantee the correct orientation of each joint, ensuring that the joint axes were properly aligned (Figure 3b). Next, the fish body model was selected, and, while holding the Shift key, the root joint of the Advanced Skeleton chain was chosen to bind the model to the skeleton. Within the weight adjustment tool, various joints were selected, and the brush tool was employed to modify their influence areas, ensuring that each joint controlled the model’s deformation within a reasonable range by adjusting the weights, thereby preventing unnatural stretching or distortion. Subsequently, the auto-generated controllers feature in Advanced Skeleton was utilized to create controllers for the skeletal chain (Figure 3c). Finally, the positions of the controllers were adjusted to ensure that each major joint had a corresponding controller for animation and deformation purposes (Figure 3d) [38].

#### 2.2.5. Animation Creation

**Figure 4:**
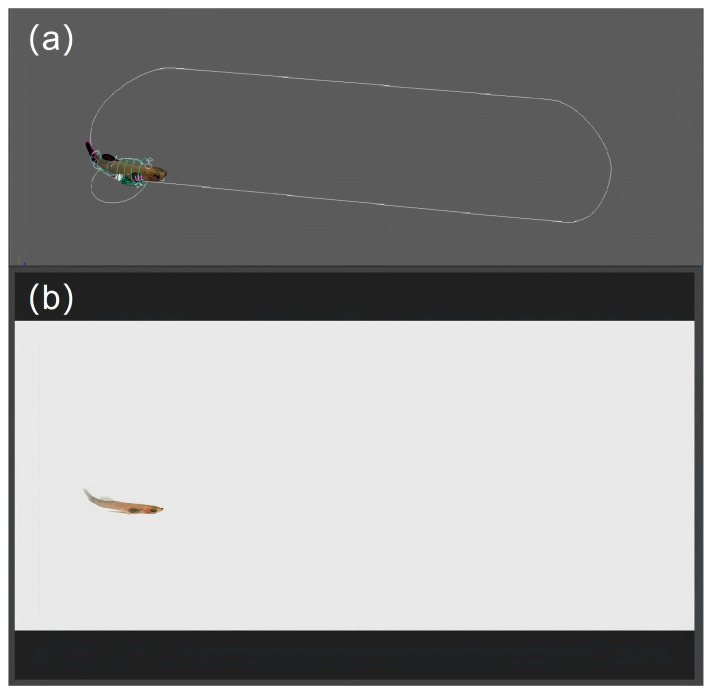
Animation creation. (**a**) Insert keyframes. (**b**) Lighting.

In Maya, the animation frame range was established on the timeline. This study required a 10 s animation at a frame rate of 24 frames per second (fps), leading to a timeline spanning from 0 to 240 frames. Keyframes were then positioned on the timeline to denote the controller’s position, rotation, and scale. The timeline slider was moved to the midpoint of the animation (frame 120), where the controller’s position and rotation were adjusted to create the fish’s intermediate pose, followed by the placement of another keyframe. Subsequently, the slider was moved to the end of the animation (frame 240), and the controller’s position and rotation were again modified to finalize the fish’s pose, after which another keyframe was set. The animation was previewed in Maya, and the keyframes and animation curves were further refined to ensure that all actions and transitions met the intended expectations (Figure 4a). Arnold was selected as the renderer, and a spherical light source was incorporated into the scene to ensure the adequate lighting of the fish (Figure 4b). The render resolution, frame range (0–240 frames), and output format were configured. After confirming that all materials and textures were correctly applied and displayed in the render, the rendering of the entire animation sequence was initiated. Upon the completion of rendering, the output frame sequence was imported into the video editing software (Adobe Premiere Pro 2020) for compositing and post-production [38].

### 2.3. Mate Choice Test

At 24 h before the mate choice experiment, we randomly selected adult test fish from the holding tanks and segregated them by sex into 96 L tanks for isolation. To mitigate aggressive interactions, each fish was housed individually in a 1.5 L clear perforated plastic bottle, allowing for water exchange with the environment [39]. The mate choice test employed a dichotomous association preference test, with the setup and procedure adapted from Chen et al. [37]. The experimental apparatus comprised a glass tank measuring 60 × 35 × 40 cm and two 22-inch computer screens (E2216H, Dell, Guangzhou, China) positioned on the short sides of the tank. To ensure uniformity in the display, both screens were calibrated to identical brightness and color settings before the experiment. The choice areas within the tank were delineated with a marker, creating two 10 cm preference zones adjacent to the screens and a central 40 cm neutral zone. The long sides of the tank were covered with gray plastic sheets to minimize external disturbances. A foam board was placed at the bottom to ensure the visibility of the screens above the water surface, with the water level maintained at 25 cm to align with the screens’ height. A high-definition camera (Logitech C920, Beijing, China) was mounted approximately 70 cm above the tank to facilitate the remote monitoring of the experiment via a computer, thereby preventing human interference. Illumination was provided by a 35 W LED light positioned 40 cm above the tank, and an air stone connected to an air pump was placed within the tank for aeration. The water was changed after every four trials to maintain its quality. At the beginning of the test, the focal individual was placed into a transparent plexiglass cylinder (10 cm diameter), positioned at the center of the neutral zone within the tank, while the first pair of animations was played. Following a 3 min habituation period, during which the fish were able to view both animations, the cylinder was gently removed. Subsequently, a 5 min observation period was conducted, during which we recorded the association times, defined as the duration spent in each preference zone. To minimize potential side biases, we relocated the focal fish to the central cylinder, swapped the side assignments of the 3D stimulus animations, and repeated the measurement of the association preferences after an additional 3 min habituation period [37]. Additionally, the standard length (SL) of each test fish was measured. The mate choice preference was assessed by calculating the strength of preference (SOP), based on the total time spent by each fish in the preference zones; more time spent in the zone with the preferred mate indicated a stronger preference. We tested *n* = 15 females and *n* = 15 males for their mate choice preferences in each trial, i.e., a total of 45 females (mean ± SD, SL: 28.49 ± 3.30 mm) and 45 males (SL: 21.83 ± 2.90 mm). The experiment consisted of three components: (1) the validation of the effectiveness of 3D simulation animations as mate choices, utilizing both 3D animations and cylindrical objects of equivalent size and color as test stimuli; (2) the exploration of individual preferences for virtual mates based on body size differences using 3D animations, where only the body length varied between the left and right animations; (3) the investigation of individual preferences for 2D versus 3D simulation animations, employing animations of the same body length for both 2D and 3D mate choice tests.

### 2.4. Statistical Analyses

All statistical analyses were conducted in SPSS 19.0 (IBM, Armonk, NY, USA). All descriptive statistics are presented as mean ± SD values, and *p* < 0.05 indicated a statistically significant difference in the results.

We estimated each focal individual’s strength of preference (SOP) for cylindrical objects versus 3D animations, large 3D animations versus small 3D animations, and 2D animations versus 3D animations as mating partners as follows: SOP = time spent associating with preferred stimulus fish/time spent with both stimulus fish. Therefore, it can be concluded that the SOP values indicate the intensity of the experimental fish’s preferences for various animations. By comparing the magnitudes of the SOP values, we can determine the degree of individual preference for different virtual mate objects. To test for overall preferences within each group and test situation, we compared the SOP values against a null assumption (i.e., SOP = 0) by using one-sample *t*-tests. Subsequently, a generalized linear model (GLM) was used to analyze the effect of the standard length (SL) of the experimental fish on the mate preference, with the SOP value as the dependent variable, the animation number as a random factor, and the standard length (SL) of the experimental fish as a covariate [40].

## 3. Results

### 3.1. Three-Dimensional Simulation Animations

**Figure 5:**
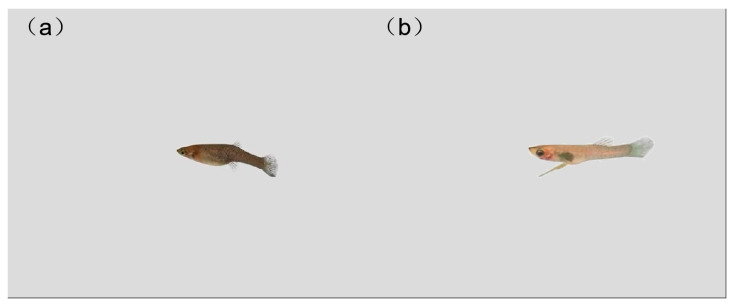
Three-dimensional simulation animation model of *G. affinis*. (**a**) Female. (**b**) Male.

A total of twenty 3D simulation animations of the *G. affinis* were created, employing the body length as a variable. These animations were organized into five groups for each sex, with each group consisting of one large and one small specimen (Figure 5a,b). The animation videos can be found in the online Supplementary Materials.

### 3.2. Validity of 3D Simulation Animations

**Figure 6:**
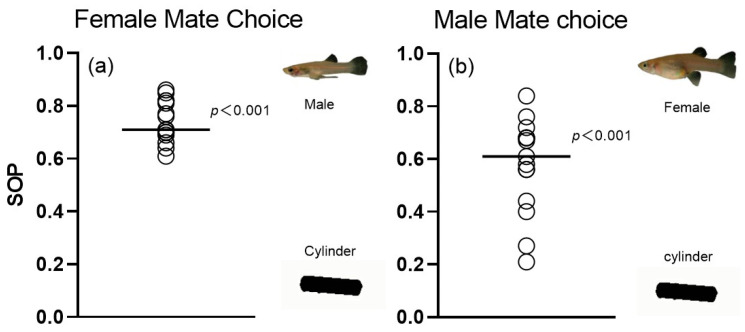
Distribution of individual strength of preference (SOP) values derived from dichotomous association preference tests. (**a**) Female choice for 3D versus cylindrical object animations. (**b**) Male choice for 3D versus cylindrical object animations. Solid lines represent the mean SOP across individuals. Results from one-sample *t*-test testing against the null assumption (SOP = 0) are presented.

The results of the GLM test indicated that the “Animation ID” was not significantly different from the “SL” when considered as a random variable and a covariate, respectively (Table 1). We initially predicted that both male and female *G. affinis* would exhibit a significant preference for the 3D virtual fish, and the results indeed confirmed our hypothesis. The results of the mate choice tests showed that, compared to the time spent in the preference zone for the cylindrical object animations (75.07 ± 35.05 s), females spent significantly more time near the 3D simulation animations (207.20 ± 38.10 s; *p* < 0.001; Figure 6a). Similarly, males also spent significantly more time with the 3D simulated animations (182.27 ± 58.30 s; *p* < 0.001) compared to males with the cylindrical object animations (137.47 ± 66.63 s; Figure 6b).

### 3.3. Overall Preferences for Mate Choice Based on 3D Simulation Animations

**Figure 7:**
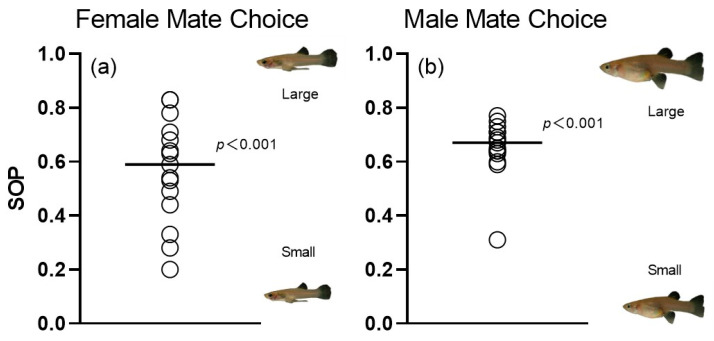
Distribution of individual strength of preference (SOP) values derived from dichotomous association preference tests. (**a**) Female choice for large versus small male 3D animations. (**b**) Male choice for large versus small female 3D animations. Solid lines represent the mean SOP across individuals. Results from one-sample *t*-test testing against the null assumption (SOP = 0) are presented.

The results of the GLM test revealed no significant differences between the random variable “Animation ID” and the covariate “SL” (Table 2). A statistically significant effect was detected in both males and females, and pronounced variation in individual preferences (i.e., SOP values) was observed. Females spent, on average, significantly more time in association with larger male 3D simulation animations (190.93 ± 63.81 s; *p* < 0.001) than with small-bodied male animations (148.20 ± 66.41 s; Figure 7a). Likewise, males also exhibited significantly more time near large-bodied female 3D animations (200.40 ± 39.07 s; *p* < 0.001) compared to males with smaller females (107.67 ± 34.89 s; Figure 7b).

### 3.4. Comparison Between 3D and 2D Simulation Animations

**Figure 8:**
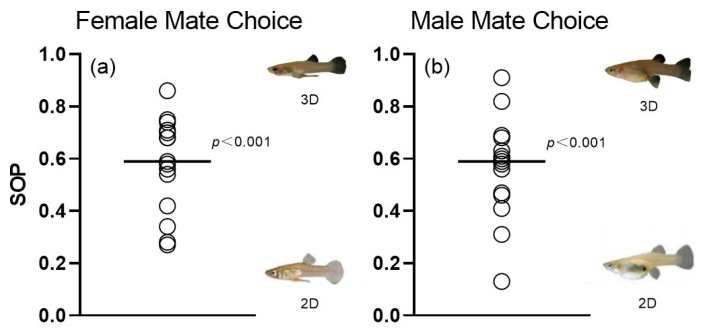
Distribution of individual strength of preference (SOP) values derived from dichotomous association preference tests. (**a**) Female choice for male 3D versus 2D animations. (**b**) Male choice for female 3D versus 2D animations. Solid lines represent the mean SOP across individuals. Results from one-sample *t*-test testing against the null assumption (SOP = 0) are presented.

The results of the GLM test revealed no significant differences between the random variable “Animation ID” and the covariate “SL” (Table 3). The results indicate that, compared to the time spent near 2D animation males (151.13 ± 68.53 s), females exhibited significantly more time with 3D simulation animations (211.00 ± 77.68 s; *p* < 0.001; Figure 8a). Meanwhile, males also showed significantly more time in association with 3D simulation animations (186.67 ± 87.03 s; *p* < 0.001) than with 2D animations (137.6 ± 66.04 s; Figure 8b).

## 4. Discussion

### 4.1. Quality of 3D Simulation Animations

We used the Maya 3D technology to produce a total of twenty 3D simulation animations of both male and female *G. affinis* in this study, with five groups for each sex. We are dedicated to investigating mate choice preferences based on 3D animation models, aiming to explore the application of 3D animation technology in the field of fish mate choice. Recently, 3D simulated animation technology has been reported in behavioral studies of reptiles due to its outstanding image processing and precise behavioral feature simulation capabilities. For instance, by employing the Maya 3D technology to examine the movement of the Jacky dragon (*Amphibolurus muricatus*) surrounded by plant noise, researchers have demonstrated the practicality of this technology in studying the visual perceptions of this species [29]. However, in the actual production process of 3D simulation animations, species differences can lead to variations in the application of skeletal binding techniques and methods [41]. This study utilized the Maya software to establish 3D animated models for *G. affinis*, and, by comparing the animation models of our study object and *A. muricatus*, we identified several key differences in the skeletal binding techniques. These differences arise from the anatomical structures and locomotion mechanisms of the two types of species. Generally, the body structures of reptiles are more complex, featuring prominent limbs, long necks, tails, and other characteristics. This skeletal structure includes multiple joints and intricate skeletal connections, particularly in the limbs and head. The accurate simulation of the movement of these parts is required during 3D modeling, as well as considering how muscles propel limb movement. In contrast, the bodies of fish are typically streamlined, primarily relying on the tail fin and other fins for swimming [42,43]. The streamlined shape of their bodies is more pronounced to facilitate rapid movement in water. Due to the simpler skeletal systems of fish, primarily consisting of vertebrae and fewer joints, this implies that 3D modeling must focus on simulating body flexion and tail fin oscillation. Therefore, this movement pattern necessitates dynamic spinal activity and tail oscillation in the 3D model, which requires the model to not only have smooth skeletal animations but also simulate the effects of water flow on the body.

The application of 3D animation technology in fish behavior research has currently received only limited reports. For example, researchers created a 3D simulation animation of the swimming Medaka (*Oryzias latipes*) using the Blender software to explore the potential application of 3D animation technology in fish behavior studies [31]. In comparison to the Medaka model created in Blender, the model produced in this study using the Maya software demonstrated superior smoothness and realism in the body and head, closely resembling the actual *G. affinis*. First, the Maya software is equipped with advanced modeling tools, making it particularly suitable for the creation of smooth biological surfaces, such as the streamlined shape of fish [44]. By precisely controlling the surface details, highly realistic 3D animation models can be produced. Secondly, the Arnold rendering engine used in this study provides exceptional light and shadow effects, optimizing the treatment of light transmission and material properties, including transparency, detailed skin textures, and color variations. Therefore, the skin texture constructed based on Maya’s 3D technology appears more natural and realistic, which are key factors in achieving lifelike fish models. Although Blender is a powerful open-source tool capable of producing high-quality animations, Maya excels in terms of its advanced features and professional support. The advantages of Maya mainly lie in its robust modeling capabilities, advanced dynamic systems, high-end material and rendering options, and extensive industry plugin support. These factors collectively ensure that Maya can produce more realistic animation effects, making it particularly prominent in creating highly lifelike fish animations and providing new potential solutions for fish behavior research.

### 4.2. Validity of 3D Simulation Animations in Fish Mate Choice

This study validated the effective recognition of 3D simulation animations by *G. affinis* based on binary mate choice tests. Additionally, the motivation for individual sexual behavior in male mate choice, represented by the continuous flaring of the reproductive fins and mating attempts, also demonstrated a valid response to the 3D simulation animations. This is consistent with the results obtained from previous observations and experiments in our laboratory [45]. Furthermore, we explored the choice preferences of individuals faced with virtual mates of different body sizes, finding consistency with our earlier experiments on mate choice in *G. affinis* using 2D simulation animations [37]. Finally, to investigate the potential advantages of 3D simulation animations in the study of fish mate choice, we compared individual mate preferences based on 2D and 3D simulation animations. The results revealed a significant preference for 3D simulation animations among individuals. This also demonstrates the strong advantages of 3D animations in studying the impact of complex visual signals on mate choice, as it can more accurately manipulate and present these signals, proving to be more effective than 2D animations. While 2D animations have advantages in terms of the production costs and the control of single features, 3D animations exhibit greater realism, multidimensional manipulation, and dynamic interaction capabilities in mate choice research [46]. These advantages enable 3D animations to accurately simulate complex behaviors in natural environments, enhancing the ecological validity and standardization levels of experiments [26]. Therefore, the application of 3D animations in mate choice research offers significant advantages, particularly in studies requiring high precision and complex behavior demonstration.

Recently, research on fish mate choice behavior based on 3D simulation technology has been reported in a study of the sailfin molly (*Poecilia latipinna*). The researchers created animations of *P. latipinna* using the FishSim animation Toolchain (FishSim Animation Toolchain, https://bitbucket.org/EZLS/fish_animation_toolchain/, accessed on 15 November 2024) and Blender v.2.70a software (Blender Foundation, Amsterdam, the Netherlands), conducting mating choice experiments by controlling the swimming behavior of animated fish with a game controller, thereby introducing new perspectives and techniques into the field of animal behavior research [33]. The advantage of this method lies in the ability to investigate individual mate selection behavior through the artificial control of fish behavior. Using animation technology to study fish mate choice through visual signals raises a key question: can individuals distinguish between live fish and 3D animations? If they can reliably identify animations and show consistent preferences for both live fish and animations, then animations could effectively replace live fish in future studies. However, comparative studies on this topic are limited, as many have only tested stimuli individually, without direct comparisons. For instance, a study on *P. latipinna* found that females responded equally to both animations and videos, and individuals showed a stronger response to 3D animations than to 3D boxes. Additionally, males could also differentiate between animations of both sexes [32]. This study demonstrated the advantages of 3D animation in mate choice research on *P. latipinna* and its potential to replace live fish. However, it did not directly compare the preferences for live fish versus animations, which is a gap in this field. While this may not significantly affect the use of 3D animations in fish mate choice studies, future research that includes direct comparisons between live fish and animation stimuli would enrich the findings and deepen our understanding of how fish phenotypic cues influence courtship behavior. Compared to animations created with FishSim and the Blender software, Maya’s 3D animations demonstrate superior precision, realistically representing the true coloration and behavioral characteristics of fish in natural environments. Therefore, it is recommended that future studies combine external controllers with the Maya 3D technology, which will not only enhance the control over fish behavior but also improve the quality of fish animation models. This technology is expected to be widely applicable in various fish behavioral experiments, not limited to mate choice studies.

We found that significant technical differences existed between 2D and 3D animations when creating animation models of *G. affinis*. The first is the display quality of the animation: 2D animations are created through hand drawing or digital drawing techniques, presenting flat images with a relatively simple visual style, limited in depth and three-dimensionality [47]. In contrast, 3D animations use 3D modeling software to create three-dimensional images, generating realistic visuals through rendering techniques. This can portray complex light and shadow effects, authentic textures, and intricate details. Secondly, regarding the fineness of behavior, the precision of 2D animations depends on the number of drawn frames and details. Although the smoothness of the animation is high, the representation of movements is constrained to the two-dimensional plane. Meanwhile, 3D animation, through skeletal rigging and motion capture technology, can more intricately simulate complex behaviors and actions, providing a stronger sense of realism [48,49]. Meanwhile, in terms of skin texture, 2D animation primarily conveys texture through color, shading, and texture drawings. Although texture effects can be enhanced through technical means, they remain inherently flat and lack realism. Meanwhile, 3D animation utilizes texture mapping and rendering techniques to simulate authentic skin textures and light effects, making the appearance of the fish more lifelike [50,51]. Overall, both 2D and 3D animations have their respective advantages and characteristics when creating animation models of *G. affinis*. Specifically, 2D animation is more advantageous in terms of the production cost and efficiency, while 3D animation exhibits significant advantages in the display quality, behavioral precision, and skin texture. In the future, it is essential to select the most appropriate research method based on the specific situational needs in fish behavioral research, and the Maya 3D simulation technology is undoubtedly an excellent choice.

## 5. Conclusions

This study established an animation model of *G. affinis* using the Maya 3D technology, validating the effectiveness of 3D animation in researching mate choice in this species and exploring their mate selection patterns. Maya 3D animation holds great promise for the study of fish mate choice, as its powerful animation and simulation tools enable researchers to accurately simulate visual signals such as the body posture, coloration, and movement patterns of fish, creating highly realistic virtual fish models. This technology aids in controlling variables, allowing the testing of different visual stimuli on mate choice behavior within a virtual environment, thus providing new means to understand the mechanisms of sexual selection in fish. Furthermore, Maya can simulate complex underwater environments and lighting conditions, making experiments more closely aligned with natural scenarios, thereby enhancing the validity and ecological significance of the experimental results. In future research, we will focus on more efficiently producing 3D simulation animations of fish using deep learning technologies and will conduct further exploration in scene rendering.

## Figures and Tables

**Table 1:** Results of generalized linear model (GLM) testing for the effects of the chosen animation type (3D/cylindrical object animations) of the individual on the strength of female and male preferences.

Sex	Factor	*df*	*F*	*p*	Wilks’ Partial *np*^2^
Female	Animation ID	10	0.40	0.88	0.02
	SL	1	1.42	0.29	0.06
	Error	15			
Male	Animation ID	10	0.93	0.51	0.03
	SL	1	0.03	0.87	0.001
	Error	15			

**Table 2:** Results of generalized linear model (GLM) testing for the effects of the choosing individual’s body size (SL) on the strength of female and male preferences.

Sex	Factor	*df*	*F*	*p*	Wilks’ Partial *np*^2^
Female	Animation ID	10	1.22	0.40	0.04
	SL	1	2.31	0.17	0.08
	Error	15			
Male	Animation ID	10	1.10	0.48	0.03
	SL	1	0.001	0.97	2.83
	Error	15			

**Table 3:** Results of generalized linear model (GLM) testing for the effects of the chosen animation type (3D/2D animations) of the individual on the strength of female and male preferences.

Sex	Factor	*df*	*F*	*p*	Wilks’ Partial *np*^2^
Female	Animation ID	10	1.11	0.48	0.04
	SL	1	0.02	0.90	0.001
	Error	15			
Male	Animation ID	10	1.82	0.24	0.05
	SL	1	4.12	0.09	0.11
	Error	15			

## Data Availability

The original contributions presented in the study are included in the article; further inquiries can be directed to the corresponding author.

## Supplementary Materials

The following supporting information can be downloaded at: https://www.mdpi.com/article/10.3390/ani14233369/s1, Figure S1: A map showing the distribution of the sampling sites in Longyan City, Fujian Province, China; Figure S2: Images of (a) males (M1–M10) and (b) females (F1–F10) used to generate 3D simulation animations; Video S1: Male 3D simulation animation; Video S2: Female 3D simulation animation.
