# Supplementary material for: Mate Choice in Western Mosquitofish (Gambusia affinis) in Response to Virtual Mates: A Method for the Investigation of Fish Mate Choice Using Maya 3D Simulation Technology
Source: Animals (Basel). 2024 Nov 22;14(23):3369. doi: 10.3390/ani14233369 (PMC11640169; doi:10.3390/ani14233369)
Supplement: Supplementary file 1 [file animals-14-03369-s001.zip › Supplementary Materials.pdf]

Supplementary Materials

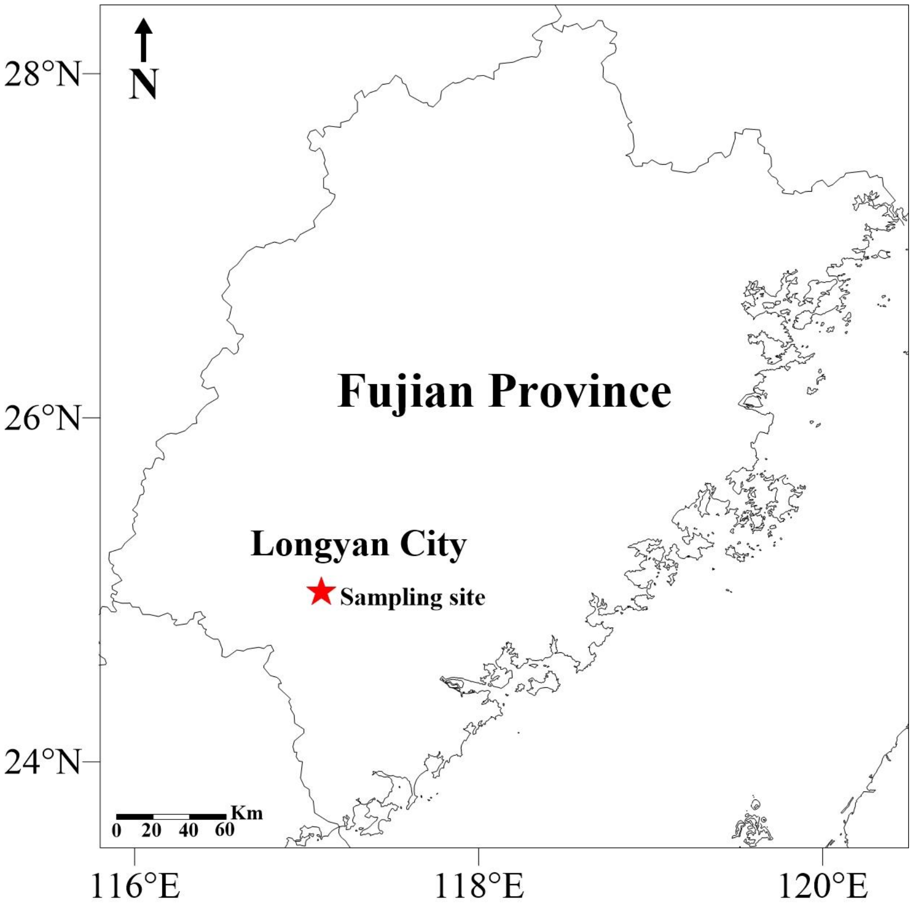

Figure S1. A map showing the distribution of sampling site of Longyan City, Fujian Province, China.

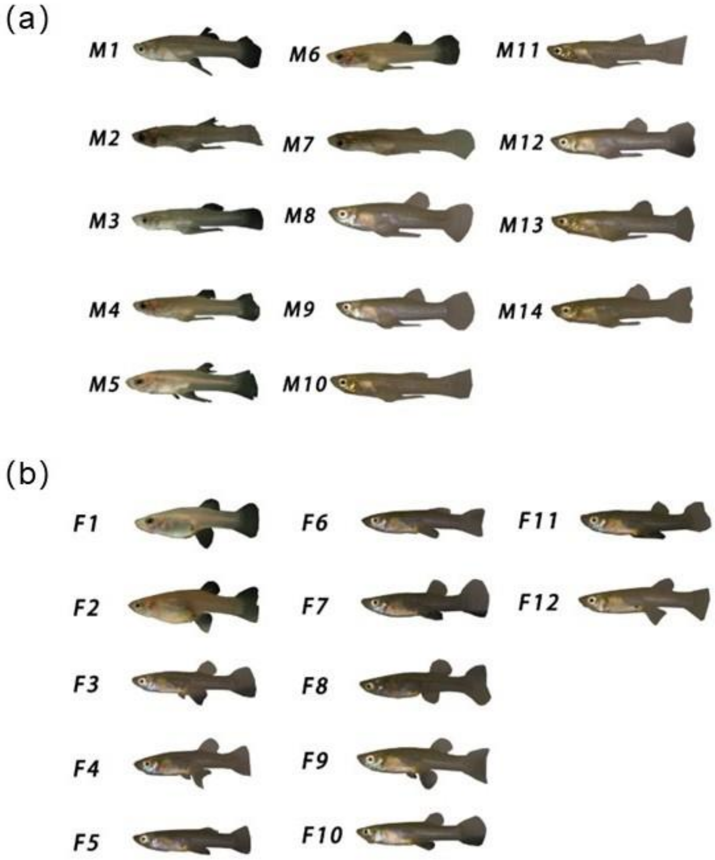

Figure S2. Images of (**a**) males (M1 –M10) and (**b**) females (F1 –F10) used to generate 3D simulation anima-tions.
